# Stenting of Malignant Urinary Tract Obstructions in Humans and Companion Animals

**DOI:** 10.3390/vetsci9010013

**Published:** 2021-12-30

**Authors:** Carrie A. Palm, Noah E. Canvasser, Willian T. N. Culp

**Affiliations:** 1 Department of Medicine and Epidemiology, School of Veterinary Medicine, University of California-Davis, Davis, CA 95616, USA; 2 Department of Urologic Surgery, University of California-Davis Medical Center, Sacramento, CA 95817, USA; ncanvasser@ucdavis.edu; 3 Department of Surgical and Radiological Sciences, School of Veterinary Medicine, University of California-Davis, Davis, CA 95616, USA

**Keywords:** stent, ureter, urethra, neoplasia, companion animals

## Abstract

Urine retention secondary to neoplastic obstructions of the upper and lower urinary tracts is a life-threatening condition in both humans and companion animals. Stents can be placed to temporarily or permanently open obstructed urinary tract lumens and are often able to be placed using minimally invasive techniques with guidance via ultrasonography or fluoroscopy. The literature for these techniques is vast for humans and growing for companion animals. The below review provides a discussion of the principles of stenting and types of ureteral and urethral stents, as well as the techniques for placing these stents in humans and companion animals.

## 1. Introduction

Both intrinsic and extrinsic neoplastic disease can result in urinary tract obstruction in humans and companion animals. Malignancy more commonly results in obstruction of the ureter than the urethra in humans, but the opposite is true in companion animals. Stenting of lower urinary tract tumors in dogs is one of the most commonly performed interventional oncology procedures in veterinary patients, and the literature descriptions of outcomes associated with urethral stenting are increasing. While stenting of urinary tract obstructions is essentially a palliative procedure to reestablish urine flow, the use of stents for delivery of local therapeutics is growing.

## 2. Principles and Concepts of Stenting

Interventional oncologic techniques are focused on the use of image guidance to perform procedures in a minimally invasive fashion. In the urinary tract, stents are commonly used to provide minimally invasive treatment options for malignant obstructions. A stent is a tube-like structure that can be implanted to allow for the temporary or permanent opening of a lumen. In the urinary tract, the goal of stenting is to reestablish urine flow allowing the excretion of urine from the body.

The imaging modalities most utilized for stent placement are endoscopy, ultrasound, and fluoroscopy. When benign ureteral obstructions are encountered, endoscopic assistance is used regularly to place ureteral stents in humans and companion animals; however, malignant tumor growth over the ureterovesicular junction (UVJ) in companion animals often limits the usefulness of direct visualization of the UVJ, and percutaneous access to the kidney with ureteral stent placement in an antegrade fashion is more commonly utilized.

Stents are commonly placed over a guidewire, although some can be passed through a catheter or sheath which has been sized to allow the desired stent to fit. Dilation of the obstructed region may be necessary using serial dilators (or dilator/sheath combinations) or via balloon dilation [1]. Some stents, particularly those placed in the urethra, can generate an outward force after placement due to a self-expanding nature, in which case pre-dilation may not be required.

## 3. Types of Urinary Tract Stents

### 3.1. Ureter

In humans, indwelling stents have been the preferred treatment for ureteral obstruction for many years as the alternative is an externalized percutaneous nephrostomy tube. Although both tubes are temporary, nephrostomy tube exchange is typically performed every 2–3 months, while stents can remain in place for up to 12 months. Ideally, ureteral stents should be rigid enough to allow for antegrade urine flow from the kidney to the bladder but pliable enough to prevent pain and be well-tolerated by the patient. In humans, ureteral stents are often uncomfortable, with a significant negative impact on quality of life. Deciding between the increased discomfort of a ureteral stent or the inconvenience of a nephrostomy bag is a common discussion with the patient prior to proceeding.

Ureteral stents often have a double-J or double-pigtail configuration and span the length of the ureter from the renal pelvis to the bladder [1,2,3]. These stents have historically consisted of a polymer tube with multiple fenestrations to allow drainage, but metallic ureteral stents have also been designed in numerous configurations. [4,5]. For these metallic stents, a metal coil may be embedded within the polymer; more recent designs are configured in a metallic spiral design. These metallic stents are designed to be magnetic resonance imaging-compatible and may be more resistant to encrustation [1,5]. Self-expanding and thermoexpandable metallic ureteral stents have also been used. These are shorter stents that only span the length of the obstruction and can be covered to help prevent tissue ingrowth [6]. While these stents have demonstrated longer patency rates compared to double pigtail stents, they do have higher rates of complications, including restenosis, migration, and can be more challenging to remove [7].

Placing two stents side by side, also called tandem stenting, can also be considered if a single stent fails [8,9]. The advantage of placing tandem stents is that the larger outer diameter of the combined stents, and the space between the two stents, allow more effective urine outflow. Experimental models suggest that the use of tandem stents may result in lower renal pelvis pressures compared with single stents [8], but further investigation is needed.

Currently, the ureteral stents most commonly utilized in canine and feline patients are made of a solid polymer along their length with a double-pigtail configuration and multiple fenestrations. In dogs and cats, neoplastic ureteral obstructions occur most commonly at the level of the trigone, secondary to lower urinary tract tumors. Given this, the continual outward radial force is generally not necessary to maintain a patent ureter. Instead, the establishment of a conduit that allows for urine flow from the kidney to the bladder is the goal. There is evidence that ureteral dilation occurs secondary to ureteral stent placement [10]; however, it is unknown if this occurs for neoplastic obstructions.

### 3.2. Urethra

In humans, both permanent and temporary urethral stents have been utilized [11,12,13]. Many stents are designed to be self-expanding, with more recent updates including thermoexpandable and biodegradeable stents [14,15]. Urethral stents, however, have a high failure rate due to encrustation, restenosis, and pain and are rarely employed in lower urinary tract obstructions [15]. Most patients are managed with a urethral or percutaneous suprapubic catheter exchanged on a monthly basis.

In dogs, both temporary and permanent urethral stents have been successful treatment options. For neoplastic disease, the majority of the permanent stents are made of a metallic alloy, with nitinol predominating. These stents are created through a laser cutting technique, are non-reconstrainable, and do not foreshorten during deployment. When placed to treat a malignant obstruction, an uncovered version is usually chosen, as this allows the stent to engage the tumor tissue, decreasing the risk for stent migration. Permanent stents can be delivered in balloon-expandable fashion [16] or as self-expanding stents [16,17,18]. In the authors’ clinic, self-expanding stents are used exclusively, as there is concern that a balloon-expandable stent could be compressed over time secondary to extraluminal compression secondary to palpation. Self-expanding stents have the advantage that they generate a continued radial force until they reach their maximum diameter, and this can allow for the urethral lumen to increase over time as the stent expands.

In dogs and cats, temporary urethral stents are placed less frequently than permanent stents. Simple versions of temporary stents are usually made of rubber or polyurethane. Temporary stents can be placed as a bridge to permanent stenting; these temporary stents are placed until a permanent stent can be placed or as a means of determining whether stenting, in general, will allow for continued urine expulsion in an individual patient. Another potential indication for temporary stenting is in dogs when reflex dyssynergy or urethral functional outflow obstruction is suspected. In those cases, permanent stenting may not relieve urethral obstruction (as the functional obstruction can occur in variable parts of the urethra at different times), so temporary stents can be placed to determine whether stenting will be successful. In addition, temporary stents can be placed while medical management is initiated.

## 4. Stenting Procedures

### 4.1. Ureter

Ureteral obstruction related to malignant disease can occur for numerous reasons, and it is important to understand the etiology as this will help to determine technical and clinical success. Primary ureteral neoplasia is uncommon in both humans and companion animals [19]. Tumors originating in the bladder or urethra can extend to the level of the UVJ and can infiltrate into the ureteral lumen or wall [20]. Extraluminal ureteral compression can also occur from masses or lymphadenomegaly. Lastly, after radiation therapy in the region of the ureter, ureteral obstruction can result from fibrosis-induced stricture or alteration of ureteral elasticity with subsequent decreased peristaltic activity [4,5,21].

In humans, a cystoscopic-guided retrograde approach is generally attempted first with patients under general anesthesia or conscious sedation [22]. A telescope is introduced through the urethra and into the bladder to identify the UVJ. A guidewire is passed through the working channel of the telescope into the ureteral orifice; a catheter can be passed over the guidewire to perform a contrast nephroureterogram. After the location of the compression is identified, the guidewire is manipulated into the kidney. Depending on the length and width of the narrowing, serial balloon dilation can be performed. This also gives the opportunity to determine which stent type and width are most appropriate. The stent can be placed directly over the guidewire, or a sheath can be passed over the guidewire and up to the kidney to facilitate placement [9].

When stenting malignant obstructions, the technical failure rate of retrograde stent placement is higher compared to stenting benign lesions [3]. In humans, if retrograde ureteral stenting is unsuccessful or the UVJ is not visible, either a percutaneous nephrostomy tube or a percutaneous nephroureteral stent (NUS) (ureteral stent with a contiguous percutaneous drain) is typically placed. This ensures relief of the acute obstruction prior to placing a stent alone. With a nephrostomy tube, subsequent conversion to a percutaneous NUS is often considered to ensure the obstruction can be bypassed. Once a percutaneous NUS is in place, capping the drain port and monitoring for signs of ureteral obstruction confirms appropriate internal drainage.

In humans, a percutaneous nephrostomy, NUS, or ureteral stent is placed in the prone position. Ultrasound guidance is used to obtain needle access to a posterior calyx. While an inferior pole calyx is typically targeted to avoid the intercostal spaces, this is not always possible, and a middle or upper calyx may be punctured. After needle access, a nephroureterogram is performed, and a guidewire is inserted into the kidney. The tract is then dilated to allow the placement of a directional catheter, which helps to manipulate the wire down the ureter and into the bladder. If a percutaneous NUS is intended, this can now be passed over the guidewire and into position. If a ureteral stent alone is planned, placement of a safety wire is imperative to prevent loss of access. Once a second wire is in place, a sheath is placed over the guidewire to facilitate stent placement. The suture should be kept on the proximal end of the stent until the stent is appropriately positioned to facilitate easy removal and repositioning. Once appropriate bladder and renal curls on the stent are achieved, removal of the suture and safety wire are performed carefully to prevent stent dislodgement.

**Figure 1:**
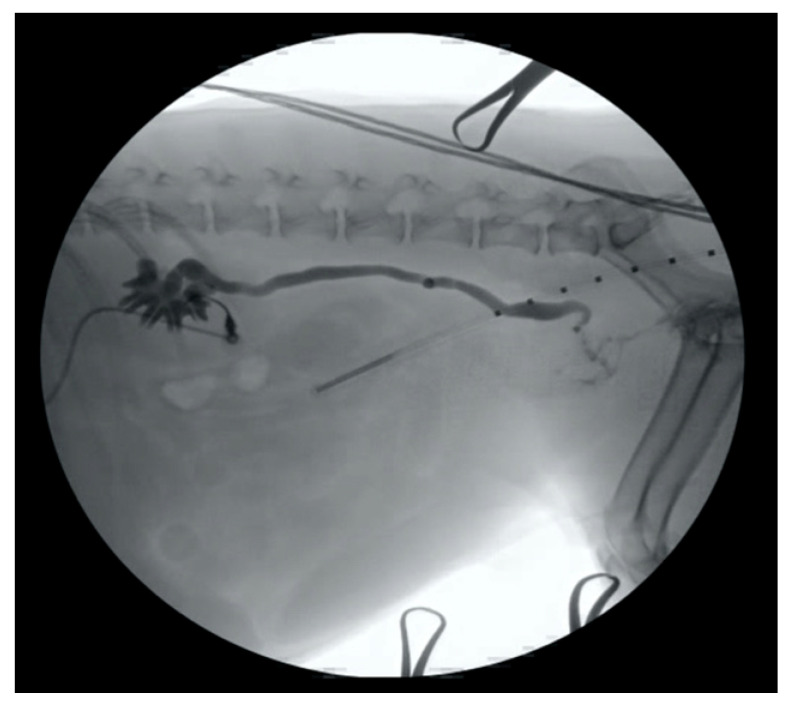
An 18-gauge over-the-needle catheter has been inserted into the renal pelvis of a dog in lateral recumbency, and a contrast nephroureterogram has been performed using fluoroscopic guidance.

**Figure 2:**
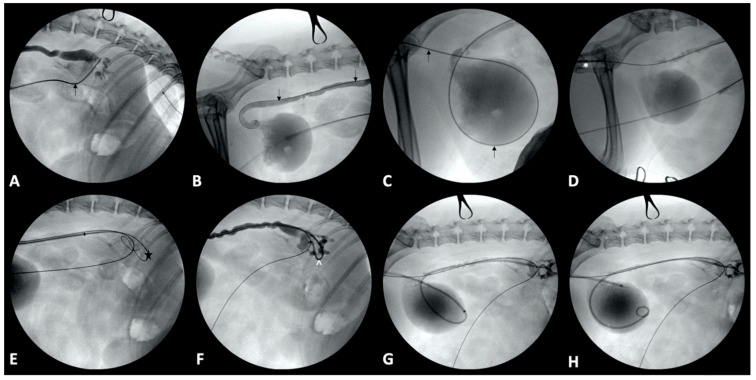
Percutaneous ureteral stent placement in an 8-year-old female spayed beagle. (**A**): A 0.035-inch × 260 cm long hydrophilic guidewire (black arrow) has been introduced into the renal pelvis through the catheter. (**B**,**C**): The guidewire (black arrow) has been manipulated down the ureter (**B**), into the bladder, and then further into the urethra (**C**). (**D**): A 6 French dilator/sheath combination (*) has been introduced into the urethra over the guidewire. (**E**): A second 0.035-inch guidewire (star) has been placed through the 6 French sheath into the renal pelvis. (**F**): A 6 French double pigtail ureteral stent (^) has been introduced through the 6 French sheath over the guidewire and into the renal pelvis. (**G**): The guidewire has been removed, allowing the cranial pigtail of the stent to curl in the renal pelvis. (**H**): The sheath has been slowly removed off the stent and utilized to gently push the caudal pigtail into the bladder.

In companion animals, general anesthesia is required for ureteral stenting, and in the author’s clinic, ureteral stent placement for malignant obstructions is performed almost exclusively via a percutaneous antegrade technique (Figure 1 and Figure 2). Companion animals are placed in lateral recumbency with the affected side up. Ultrasound guidance is utilized to introduce an 18-gauge over-the-needle catheter into the renal pelvis, ideally with access directed towards the ureteropelvic junction (UPJ). After removing the needle from the catheter, a T-port is attached to the catheter, and a 50%/50% mixture of iodinated contrast/saline is injected to perform a nephroureterogram. Once the renal pelvis and ureter have been delineated, the region of obstruction is identified, and the T-port is disconnected from the catheter. If the catheter has been positioned towards the UPJ, guidewire passage into the ureter from the renal pelvis is often simplified. A 0.035-inch × 260 cm long hydrophilic guidewire is introduced into the renal pelvis through the catheter. Fluoroscopic guidance is used to manipulate the guidewire down the ureter and into the bladder. The guidewire is then passed out of the urethra to exit the urethral orifice and is grasped on both ends (“through-and-through” guidewire access). A 6 French dilator/sheath combination is introduced into the urethra over the guidewire. The dilator and sheath are passed into the ureter, and the dilator is removed. A second 0.035-inch guidewire is then placed through the 6 French sheath retrograde into the renal pelvis. The 6 French sheath is removed off the through-and-through guidewire, the dilator is reintroduced into the sheath, and the combination is passed over the guidewire in the renal pelvis. The dilator is removed, and a 6 French (medium to large dogs) or 4.7 French (small dogs) double pigtail ureteral stent is introduced through the 6 French sheath over the guidewire and into the renal pelvis. The guidewire is removed, allowing the cranial pigtail of the stent to curl in the renal pelvis. The sheath is slowly removed off the stent and utilized to gently push the caudal pigtail into the bladder. After confirmation of appropriate placement, all guidewires and the sheath are removed.

If guidewire passage through the UVJ is not possible in dogs after antegrade passage from the renal pelvis, a surgical approach for ureteral stent placement can be considered. To place a ureteral stent in a dog when the guidewire cannot be passed through the UVJ, an 18-gauge over-the-needle catheter is passed through the dilated ureter from a point cranial to the obstruction, then through the tumor at the level of the UVJ, into the bladder and finally out of the bladder so that the hub of the catheter is on the ureter side, and the catheter tip is out of the bladder. A 0.035-inch guidewire is then passed into the tip of the catheter and out of the hub of the catheter. The catheter is withdrawn out of the ureter so that through-and-through guidewire access is achieved. The guidewire is then pulled caudally until the tip is in the ureter, after which it is then advanced into the renal pelvis. A sheath with a dilator is advanced over the guidewire (a small incision into the bladder large enough to allow sheath passage may be necessary), through the ureteral obstruction and into the renal pelvis, and the dilator is removed. The ureteral stent is then delivered over the guidewire, through the sheath, until the cranial pigtail is located in the renal pelvis. The sheath is removed. The guidewire is subsequently removed, and the caudal pigtail is passed through the opening that had been created in the bladder by the sheath. If a hole large enough for urine leakage is present in the bladder wall, a suture should be placed for closure.

In cats, ureteral stenting of malignant obstructions is performed as described above after a celiotomy with the cat positioned in dorsal recumbency. Due to the challenging caudal location and small size of the feline UVJ, a cystotomy (and possibly cranial urethrotomy) may be necessary to gain access to the guidewire after passage through the UVJ.

Complications of ureteral stenting may include ureteral or vascular injury, local tissue reaction to the stent with subsequent tissue overgrowth, vesicorenal reflux, stent encrustation, pyelonephritis, bladder irritation, migration, and compression resulting in reobstruction [1,3,21]. Technical success (often defined as placement of the stents using an interventional technique) is generally considered very high, with most percentages being >94% in humans [2,3]. Ureteral stents appear to be better tolerated in companion animals and rarely require exchange when placed for malignancy. In one study evaluating outcomes associated with percutaneous antegrade ureteral stenting for malignancy in dogs, the technical success rate was 100%, and all dogs demonstrated an improvement in azotemia prior to discharge from the hospital [20]. Additionally, in the 10 dogs that underwent post-stenting ultrasonographic examination, improvement in hydronephrosis and ureteral dilation was noted [20].

### 4.2. Urethra

Urethral stent placement in humans is rare as the alternative treatment options for malignant obstructions of the lower urinary tract are often considered more tolerable [12,13]. Additionally, the results of urethral stent placement in humans are questionable as complications can be severe, and removal is often challenging or impossible [11,12,13].

Lower urinary tract tumors are relatively common in dogs and can cause significant morbidity [23]. By far, the most common lower urinary tract tumor affecting both dogs and cats is transitional cell carcinoma (urothelial carcinoma) [24,25]. While metastasis of urothelial carcinoma occurs regularly, urethral obstruction from the primary tumor is most often the life-limiting factor as dogs and cats can lose the ability to pass urine or experience significant stranguria and/or overflow incontinence from bladder obstruction.

Urethral stents in companion animals can be placed using image guidance by accessing the natural orifice of the urethra, with the one exception being male cats who have not undergone a perineal urethrostomy. In those male cats, the diameter of the urethral orifice is too small to allow passage of the delivery system of the urethral stent in a retrograde fashion, and a small surgical approach to the bladder is necessary for passing instrumentation into the urethra and performing stent placement in an antegrade fashion. In male and female dogs, female cats, and male cats who have had a perineal urethrostomy, the animal is placed in lateral recumbency. The use of fluoroscopy is ideal as it allows for real-time alterations to the procedure and assessment of anatomy; however, urethral stents can be placed with digital radiography as well [26]. The vulva or prepuce are clipped free from hair, prepared with aseptic technique, and draped.

**Figure 3:**
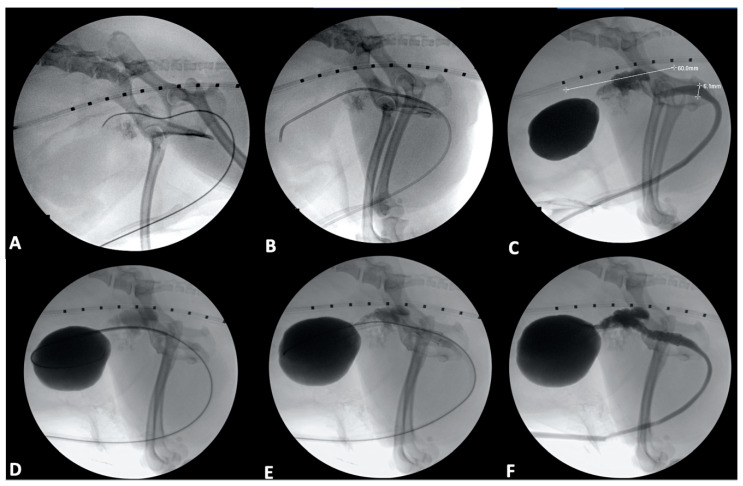
Urethral stenting in a 9-year-old male castrated toy poodle. (**A**): A 0.035-inch hydrophilic guidewire has been passed into the urethra and further into the trigone of the bladder. (**B**): A 4 French angled catheter has been introduced over the guidewire and advanced into the bladder. (**C**): A contrast cystourethrogram has been performed, and initial measurements of the region of the urethra affected by tumor have been obtained (white line extending cranial to caudal), as has a urethral diameter (white line extending dorsal to ventral). Note the infiltration of contrast into the prostate through the prostatic ductules. (**D**): A stent has been introduced into the bladder over the guidewire. (**E**): The stent has been deployed. (**F**): The angled catheter was passed over the guidewire, and a contrast cystourethrogram was performed to confirm patency of the urethra in the region of the stent.

In male dogs, access to the urethra is generally straightforward after extrusion of the penis from the prepuce and the introduction of a 0.035-inch hydrophilic guidewire into the urethra (Figure 3). After placement of the guidewire, a 6–8 French vascular access sheath and a dilator is placed over the guidewire. The dilator is removed, and a 4 French angled catheter is introduced over the guidewire, through the sheath, and advanced into the bladder. The bladder is filled with a 50%/50% mixture of iodinated contrast/saline through the catheter. The guidewire is removed, and a cystourethrogram is performed by pulling the angled catheter out of the bladder and along the entire length of the urethra under fluoroscopic guidance; the access sheath must be removed during the urethrogram to ensure visualization of the entire urethra. The urethrogram can also be performed through the vascular access sheath; however, luminal distention may not be as complete as compared to the use of an angled catheter. Measurements of the region of the urethra affected by the tumor are obtained, as is the urethral diameter. A stent is chosen based on the measurements of the urethral diameter and the region of stricture, allowing for additional coverage of 1 cm on each side of the stricture. The guidewire is then reintroduced into the bladder, and the vascular access sheath is replaced and sutured to the prepuce, provided that the area of obstruction is not in this distal urethra. The stent is introduced through the sheath and into the bladder over the guidewire, and the stent is deployed. The angled catheter is placed over the guidewire, and a contrast cystourethrogram is performed to confirm patency of the urethra in the region of the stent (Figure 3). Once appropriate stent positioning is confirmed, all equipment is removed.

In female dogs and cats, a guidewire is introduced into the vulva and directed toward the urethral orifice. If access into the urethral orifice is challenging, cystoscopic guidance can be utilized to facilitate guidewire placement. A 6–8 French vascular access sheath is placed over the guidewire and advanced into the urethra. Using the side-arm of the sheath, the bladder is filled with a 50%/50% mixture of iodinated contrast/saline. A contrast cystourethrogram is then performed by injecting into the sheath while pulling the sheath from the bladder and out of the urethra; the guidewire is left in position within the bladder during the procedure. The rest of the procedure is performed as described above for male dogs.

In four studies of urethral stenting for malignancy in dogs (Table 1), outcomes of stenting in 94 primary lower urinary tract tumors have been described and include 92 cases of carcinoma, one prostatic osteosarcoma, and one bladder hemangiosarcoma [16,17,18,26]. Both technical success (successful placement of a urethral stent) and clinical success (resolution of obstruction) rates are high at >94% in the described canine cases [17,18,26]. Most commonly, complications have included incontinence, reobstruction, stranguria, and stent migration [16,17,18]; one study determined that stent length, diameter, and location did not affect the incidence of incontinence [17]. Due to the findings of prolonged survival with the administration of non-steroidal anti-inflammatory medications and chemotherapy [17], further investigation is warranted into the combination of urethral stenting and anti-proliferative therapy as well as the use of prostatic artery embolization [27].

## 5. Other Indications and Advances in Urinary Tract Stenting

Stents can be designed to provide local delivery of a bioactive agent, and this is commonplace in vascular and cardiac interventions. This concept has also been investigated for use with ureteral and urethral stents as well. These can include drug-eluting stents which release these agents locally in a controlled manner or drug-coated stents, which are covered with pharmaceutical agents that provide additional characteristics to the stent [21]. The pharmaceutical agents that have been most investigated include antibiotics, anti-inflammatories, and anti-proliferative agents such as paclitaxel [21].

Local delivery of radiation therapy via stents is also being evaluated. Recently, a technique for delivery of brachytherapy from a ureteral stent was described in normal beagle dogs [27]. The ureteral stents used in that study were designed with a double cavity to allow for both the drainage of urine as well as implanting iodine-125 seeds [27]. These stents were successfully placed and determined to be safe; however, further investigation in clinical cases is needed [28].

Urethral stents have also been placed as fiducial markers to guide radiation therapy and allow for greater doses during image-guided radiotherapy [29,30]. In one study evaluating men with prostate cancer, a removable stent was placed via the urethra 2–3 weeks prior to the initiation of radiation therapy [29]. The stent was utilized to guide radiation therapy and then removed three months after treatment. Ninety percent of men completed the full treatment course, and no stent migration was reported [29]. A follow-up study performed in the same group of men five years later demonstrated that the precision of the prostatic stent as a fiducial marker was at least as good as other described techniques, and overall survival was 85% [30].

Stent technology is constantly evolving, and the hope is that modifications and advancements will allow for decreased complications associated with stents and better tolerance of stents in terms of comfort and local tissue acceptance. Additionally, as stated above, stents provide the unique advantage of being in direct contact with a lesion, and therefore, if local delivery of substances from the stents can be better established in veterinary patients, this may allow for improved outcomes.

## 6. Conclusions

Urinary tract stenting is a highly effective and minimally invasive means of reestablishing urine flow in both companion animals and humans. The technology associated with these stents is regularly advancing and includes tremendous potential for local delivery of bioactive substances. There is significant translational potential between human and veterinary medicine, and future investigation regarding how advancements in stents can improve the quality of life in all species is warranted.

## Figures and Tables

**Table 1:** Summary table of major studies evaluating urethral stent placement in dogs.

First Author	Number of Dogs Undergoing Stenting for Malignancy	Stent Type	Technical Success (Placement of Stent)	Clinical Success (Resolution of Obstruction)	Complications
Weisse C [16]	12	Self-expanding (*n* = 9), Balloon-expandable (*n* = 3)	100% (12/12)	100% (12/12)	incontinence, reobstruction, displaced stent, stranguria
Blackburn AL [17]	42	Self-expanding (*n* = 42)	100% (42/42)	98% (41/42)	incontinence, reobstruction, displaced stent, stranguria,
McMillan SK [18]	19	Self-expanding (*n* = 18; 1 dog not able to be stented)	95% (18/19)	100% (17/17) (1 dog euthanized in hospital prior to discharge: long-term outcome unable to be evaluated)	incontinence, reobstruction, displaced stent, stranguria,
Radhakrishnan A [26]	22	Self-expanding (*n* = 22)	100% (22/22)	100% (22/22)	incontinence, reobstruction, displaced stent, stranguria, guidewire passage through urethral wall
